# The LiaFSR-LiaX System Mediates Resistance of Enterococcus faecium to Peptide Antibiotics and to Aureocin A53- and Enterocin L50-Like Bacteriocins

**DOI:** 10.1128/spectrum.00343-23

**Published:** 2023-05-23

**Authors:** Aleksandra Tymoszewska, Marlena Szylińska, Tamara Aleksandrzak-Piekarczyk

**Affiliations:** a Institute of Biochemistry and Biophysics, Polish Academy of Sciences (IBB PAS), Warsaw, Poland; University of Florida College of Dentistry

**Keywords:** antibiotic resistance, aureocin A53- and enterocin L50-like bacteriocins, bacteriocin resistance, daptomycin, gramicidin, LiaFSR stress-response regulatory system, LiaX daptomycin-sensing protein

## Abstract

Multidrug-resistant Enterococcus faecium strains are currently a leading cause of difficult-to-treat nosocomial infections. The emerging resistance of enterococci to last-resort antibiotics, such as daptomycin, prompts a search for alternative antimicrobials. Aureocin A53- and enterocin L50-like bacteriocins are potent antimicrobial agents that form daptomycin-like cationic complexes and have a similar cell envelope-targeting mechanism of action, suggesting their potential as next-generation antibiotics. However, to ensure their safe use, the mechanisms of resistance to these bacteriocins and cross-resistance to antibiotics need to be well understood. Here, we investigated the genetic basis of E. faecium’s resistance to aureocin A53- and enterocin L50-like bacteriocins and compared it with that to antibiotics. First, we selected spontaneous mutants resistant to the bacteriocin BHT-B and identified adaptive mutations in the *liaFSR*-*liaX* genes encoding the LiaFSR stress response regulatory system and the daptomycin-sensing protein LiaX, respectively. We then demonstrated that a gain-of-function mutation in *liaR* increases the expression of *liaFSR*, *liaXYZ*, cell wall remodeling-associated genes, and hypothetical genes involved in protection against various antimicrobials. Finally, we showed that adaptive mutations or overexpression of *liaSR* or *liaR* alone results in cross-resistance to other aureocin A53- and enterocin L50-like bacteriocins, as well as antibiotics targeting specific components of the cell envelope (daptomycin, ramoplanin, gramicidin) or ribosomes (kanamycin and gentamicin). Based on the obtained results, we concluded that activation of the LiaFSR-mediated stress response confers resistance to peptide antibiotics and bacteriocins via a cascade of reactions, eventually leading to cell envelope remodeling.

**IMPORTANCE** Pathogenic enterococci carry virulence factors and a considerable resistome, which makes them one of the most serious and steadily increasing causes of hospital epidemiological risks. Accordingly, Enterococcus faecium is classified into a top-priority ESKAPE (Enterococcus faecium, Staphylococcus aureus, Klebsiella pneumoniae, Acinetobacter baumannii, Pseudomonas aeruginosa, and Enterobacter species) group of six highly virulent and multidrug-resistant (MDR) bacterial pathogens for which novel antimicrobial agents need to be developed urgently. Alternative measures, such as the use of bacteriocins, separately or in combination with other antimicrobial agents (e.g., antibiotics), could be a potential solution, especially since several international health agencies recommend and support the development of such interventions. Nevertheless, in order to exploit their efficacy, more basic research on the mechanisms of cell killing and the development of resistance to bacteriocins is needed. The present study fills some of the knowledge gaps regarding the genetic basis of the development of resistance to potent antienterococcal bacteriocins, pointing out the common and divergent features regarding the cross-resistance to antibiotics.

## INTRODUCTION

Enterococcus faecalis and Enterococcus faecium are common commensal bacteria of the human gastrointestinal tract, but they are also opportunistic pathogens that have emerged as a major cause of urinary tract infections, surgical wounds, endocarditis, and nosocomial bacteremia. Moreover, they have become increasingly multidrug resistant (MDR) and resistant to high concentrations of aminoglycosides such as streptomycin, kanamycin (KAN), and gentamicin (GEN), β-lactams such as ampicillin, and the last-resort antibiotic vancomycin (vancomycin-resistant enterococci [VRE]) (1, 2). Notably, they can transfer resistance genes to other medically important microorganisms, as has been shown for vancomycin-resistant Staphylococcus aureus (3). Thus, finding new antimicrobial agents against the enterococci is critically important.

The cell envelope-targeting mechanism of action is particularly promising for the development of such next-generation antimicrobials. The cytoplasmic membrane and cell wall are essential and highly conserved structures, so the development of resistance to agents targeting them is difficult (4). Daptomycin (DAP) and gramicidin (GRA) are U.S. Food and Drug Administration (FDA)-approved cell envelope-active antibiotics from the group of nonribosomally synthesized antimicrobial peptides (AMPs) (5, 6). Gramicidin forms dimeric channels in the membrane mediating ion conduction (6). DAP-Ca^2+^ oligomerizes and forms tripartite complexes with phosphatidylglycerol and undecaprenyl-coupled cell envelope precursors, such as lipid II, blocking cell wall synthesis. Complex formation triggers delocalization of several membrane-associated enzymes involved in peptidoglycan biosynthesis and drastic rearrangement of the cytoplasmic membrane (7, 8). DAP is used to treat infections caused by VRE, but despite its membrane-directed mechanism of action, DAP resistance is increasingly observed in *Enterococcus* spp. (9).

Bacteriocins, which are ribosomally synthesized AMPs of bacterial origin, are divided into posttranslationally modified lantibiotics (class I) and unmodified nonlantibiotics (class II). Nisin is the best-characterized lantibiotic with broad-spectrum and antienterococcal activity, whose dual mode of action includes inhibition of peptidoglycan synthesis through interaction with lipid II, followed by pore formation by penetrating the cell membrane (10). Among nonlantibiotics, two families of aureocin A53 (AurA53)- and enterocin L50 (EntL50)-like bacteriocins with broad-spectrum activity against enterococci are distinguished by sharing a similar structural saposin-like fold (11, 12). It is composed of three or four amphiphilic α-helices, of which the cationic, hydrophilic residues are exposed on the surface and the hydrophobic ones are packed inside to form a hydrophobic core (13, 14). The saposin-like fold is thought to enable these AMPs to penetrate the cell membrane in the absence of specific receptors and act either by pore formation or generalized displacement of membrane lipids and disruption of membrane structure (11, 14).

The resistance to membrane- or cell wall-active AMPs is often shaped by two-component stress-response regulatory systems (TCSs). LiaSR is the eponymous TCS first identified and characterized in Bacillus subtilis (15). LiaSR has been shown to respond strongly to lipid II-targeting DAP, vancomycin, or ramoplanin (RAM), as well as to many other cell envelope stress factors, such as bacitracin (BAC), nisin, cationic AMP LL-37, alkaline shock, detergents, organic solvents, secretion stress, or filamentous phage infection (16). The LiaSR system is comprised of LiaS, a bifunctional histidine kinase that acts as a kinase/phosphatase on the LiaR response regulator (RR) in the presence/absence of stressors (17). This system is genetically and functionally associated with the membrane-localized accessory protein LiaF, and together they form the three-component system LiaFSR. In the absence of a stressor, LiaF inhibits the LiaSR-dependent signal transduction (18), whereas in its presence, the phosphorylated RR induces the *liaIH-liaGFSR* locus, with the strongest activation of the *liaIH* genes encoding, respectively, a membrane anchor for LiaH and a homolog of the cytosolic phage shock protein A (PspA) of Escherichia coli (19, 20). Homologs of the LiaFSR systems are widespread among species belonging to the *Firmicutes*, with the number and nature of stressors, as well as the genes regulated by these homologs, varying widely (16). The enterococcal LiaFSR system has been studied extensively recently, mainly in the context of DAP resistance in E. faecium and E. faecalis. However, it has also been shown to be involved in resistance to other AMPs, such as lantibiotics (nisin, gallidermin, mersacidin), the antibiotic friulimicin, human beta-defensin-3 (HBD3) and cathelicidin LL-37, and synthetic RP-1 (21, 22). In E. faecalis, in addition to its own locus, LiaFSR also regulates the *liaXYZ* operon and genes for cell wall synthesis, cell division, transmembrane proteins, and cell envelope stress response (22). It has been proposed that a major modulator in this process is the surface protein LiaX, which, in the absence of DAP, acts as an inhibitor of the LiaFSR system, potentially by interacting with LiaF and LiaS, whereas the binding of DAP by LiaX results in the release of LiaFS and subsequent activation of this system (22). The increased activity of the LiaFSR system causes remodeling of the cell membrane (redistribution of anionic phospholipids away from the division septum, an increase in the content of phosphatidylglycerols, and a decrease of cardiolipin), protecting against the action of AMPs (22).

Here, we investigated the genetic basis of resistance to the AurA53- and EntL50-like bacteriocins and cross-resistance with a wide range of antibiotics in E. faecium. We isolated spontaneous resistant mutants of the sensitive E. faecium strain LMGT 2783 by exposing the cells to increasing concentrations of bacteriocin BHT-B. Single, nonsynonymous mutations in genes encoding the LiaFSR system or the LiaX protein were found in the resistant strains. Notably, these adaptive mutations caused cross-resistance to other AurA53- and EntL50-like bacteriocins, as well as to some antibiotics such as the lipid II-targeting DAP and RAM, the membrane-targeting GRA, and the positively charged KAN and GEN. The mutation in *liaR* increased the expression of *liaFSR*, *liaX*, a cell wall remodeling (*sgtB*) gene, and several hypothetical (*liaY*, *liaZ*, *xpaC*, *ef0798*, *ef0932*, *ef1533*) genes. Finally, we conducted a series of resistance assays for strains with the *liaFSR* operon or the *liaX* gene deleted or complemented. The results indicated that the resistance of the adaptive mutants is likely due to changes in the activity of individual components of the LiaFSR-LiaX (LiaFSR-X) system, resulting in the obligatory activation of LiaR, which then leads to an LiaR-dependent upregulation of the expression of genes conditioning cell envelope remodeling.

## RESULTS

### BHT-B induces the emergence of resistant mutants.

In an earlier study, we investigated the genetic basis of resistance to BHT-B, an AurA53-like family bacteriocin, in the model bacterium Lactococcus lactis (12). Here, we studied this phenomenon in E. faecium, a bacterium from a genus that includes several pathogenic and multidrug-resistant strains causing nosocomial infections (23). For this purpose, we first generated spontaneous BHT-B-resistant mutants by exposing sensitive E. faecium LMGT 2783 to increasing concentrations of BHT-B. Seven mutants with BHT-B susceptibility 4- to 16-fold lower than that of the wild-type (WT) strain (MIC_50_, 3.2 μg/mL) were obtained. They were named MUT_130 to MUT_132 and MUT_136 to MUT_139 (Table 1). For initial testing, all mutants were checked for colony morphology, growth rate in standard medium, resistance to salinity and sodium dodecyl sulfate (SDS), and stability of maintenance of the BHT-B resistance trait. Reduced sensitivity to BHT-B was maintained in all mutants for up to 10 passages, suggesting the stability of the genetic mutations acquired. Compared to WT strain E. faecium LMGT 2783, none of the mutants showed a difference in colony morphology, growth rate, or resistance to NaCl (MIC_50_, 6% ± 0.0%). Regarding resistance to SDS, all strains had similarly high susceptibilities to this detergent (MIC_50_, 0.01% ± 0.00%), with the MUT_132 mutant showing even a 2-fold increase in susceptibility (MIC_50_, 0.005% ± 0.00%).

**TABLE 1:** Spontaneous E. faecium mutants resistant to BHT-B

Mutant	Resistance to BHT-B (fold increase relative to WT)^*a*^	Mutation^*b*^	Amino acid change^*b*^
MUT_130	8*	*liaF*: 343C→T	LiaF: Gln115X
MUT_131	8*	93 nucleotides upstream of *liaX*: C→T	Not applicable
MUT_132	8*	*liaR*: 527C→A	LiaR: Thr176Lys
MUT_136	8*	*liaF*: 191delT	LiaF: Ile64IlefsX4
MUT_137	4*	*liaS*: 551A→G	LiaS: Gln184Arg
		*liaX*: 1382delT	LiaX: Leu461TrpfsX21
MUT_138, MUT_139	16*	*liaX*: 1519C→T	LiaX: Gln507X

a *, statistically significant result (*P* value < 0.05).

b →, substitution; del, deletion; fs, frameshift; X, stop codon (the number after X indicates the number of codons following that bearing the mutation, including the stop codon).

### BHT-B-resistant strains contain mutations in *liaFSR* or *liaX*.

To identify the genetic alterations responsible for the resistance to BHT-B, we sequenced the genomes of seven spontaneous BHT-B-resistant mutants and compared them with that of WT strain LMGT 2783. Seven unique single point mutations were identified in the mutants, four in the *liaFSR* operon encoding the three-component LiaFSR stress response regulatory system and three upstream (UP) of or within the *liaX* gene encoding the LiaR-regulated LiaX surface protein.

**FIG 1:**
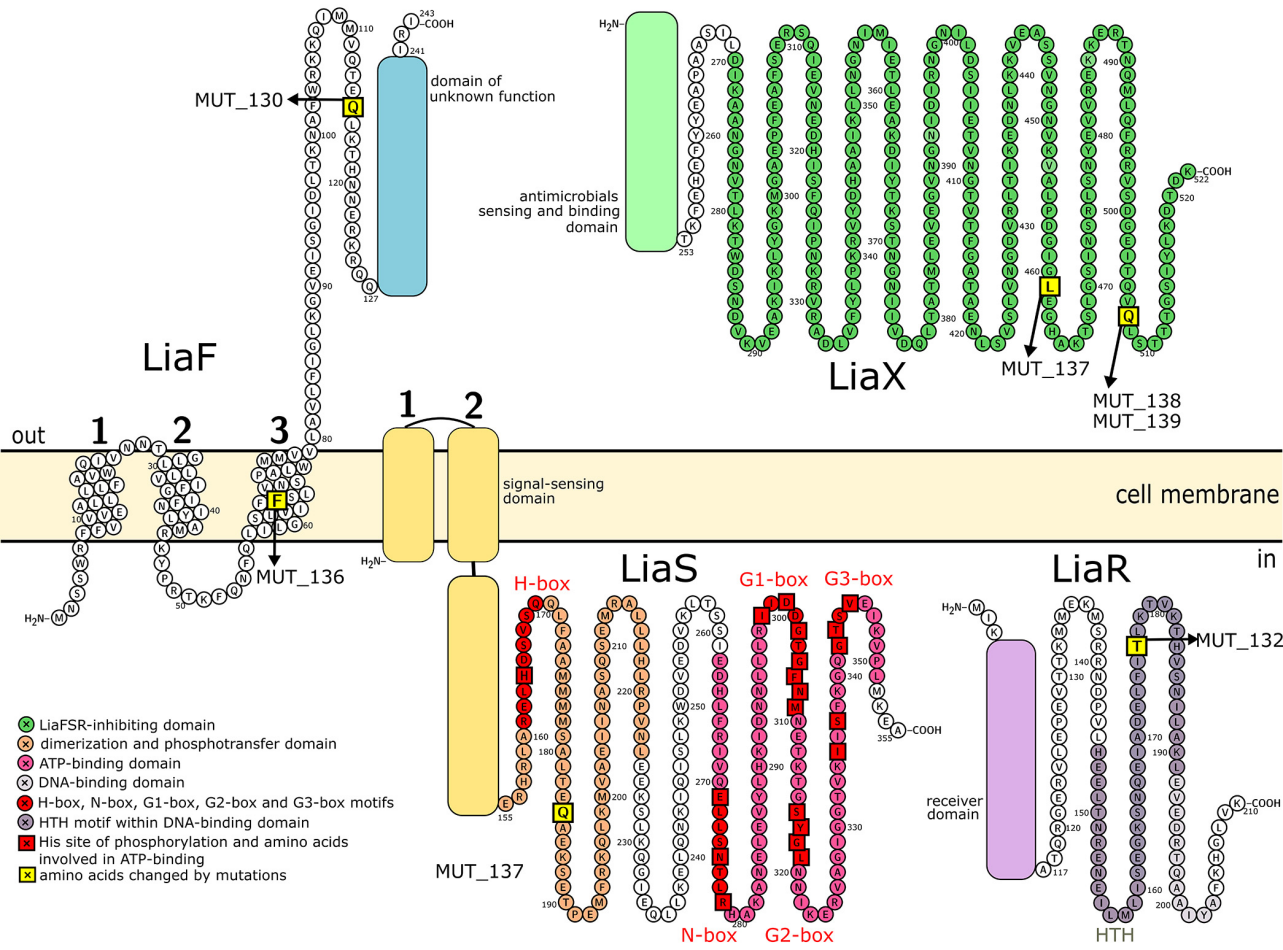
Localization of adaptive mutations in predicted topology of LiaF, LiaS, LiaR, and LiaX proteins.

E. faecium MUT_130 and MUT_136 contained a mutation in *liaF* encoding membrane-localized accessory protein LiaF (Table 1). MUT_130 had a nonsense mutation, and MUT_136 had a frameshift mutation leading to premature termination of translation of four codons downstream of that bearing the mutation; the altered amino acids were localized upstream of the LiaF C-terminal extracellular domain of unknown function (PF09922) (Fig. 1). Therefore, both mutations resulted in the production of a significantly truncated LiaF protein deprived of its C-terminal domain. MUT_132 contained a missense mutation in *liaR* causing the Thr176Lys substitution in the response regulator LiaR (Table 1). LiaR comprises an N-terminal receiver domain (PF00072) catalyzing the transfer of the phosphoryl group from the histidine residue of histidine kinase to its aspartate residue and a C-terminal LuxR-type DNA-binding domain (PF00196). The substituted Thr176 is localized in the helix-turn-helix DNA-binding motif of the DNA-binding domain (Fig. 1). In MUT_137, a missense mutation in *liaS* resulted in the Gln184Arg substitution in the histidine kinase LiaS (Table 1). LiaS comprises an N-terminal signal-sensing domain and a C-terminal catalytic core composed of a dimerization/histidine phosphotransfer (DHp) domain (HisKA_3; PF07730) and an ATP-binding one (HATPase_c; PF02518). Five amino acid motifs important for the catalytic activity have been identified in LiaS, such as the H-box motif (24) containing the histidine phosphorylation site in the DHp domain and the N-, G1-, G2-, and G3-box ATP-binding motifs (24) in the ATP-binding domain. Notably, the substituted Gln184 is localized near the H-box motif in the DHp domain (Fig. 1). Importantly, MUT_137 contained one more mutation, a frameshift in the *liaX* gene causing truncation of the LiaX protein (Table 1). Two other mutants, MUT_138 and MUT_139, also carried nonsense mutations in the *liaX* gene resulting in LiaX truncation (Table 1). A conserved domain (CD) search showed that LiaX contains only a C-terminal putative adhesin domain (PF13349), while experimental studies in E. faecalis have suggested that LiaX is comprised of two domains, an N-terminal domain formed largely of α-helices that binds antimicrobials, thereby activating the stress response, and a C-terminal one composed mainly of β-strands that inhibits the LiaFSR system most probably through an interaction with the membrane components LiaF and LiaS (22). Notably, both mutations identified here were located at the end of the LiaX C-terminal domain (Fig. 1) and resulted in its truncation. Finally, in MUT_131, a base substitution was found in the noncoding region upstream of the *liaX* gene (Table 1).

### Mutation upstream of *liaX* lies in the UP element and slightly increases the expression of some of the *liaXYZ* and *liaFSR* genes.

**FIG 2:**
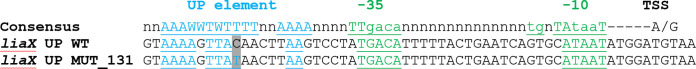
Nucleotide sequence upstream of *liaX* gene in E. faecium wild type and MUT_131. The nucleotide sequence of the *liaX* promoter in the MUT_131 strain carrying the C→T substitution (highlighted in gray) in the UP element was compared with the promoter region consensus sequence. TSS, transcription start site.

The nucleotide substitution upstream of *liaX* in MUT_131 was 12 nucleotides upstream of the −35 box of the predicted *liaX* promoter, in an A/T-rich region likely representing the so-called upstream (UP) element (25) recognized by the α subunit of RNA polymerase. The C→T substitution in a thymidine stretch increased the A/T content of the UP element from 9 to 10 (Fig. 2).

To determine the effect of the mutation in the UP element in MUT_131 on *liaX* transcription, its mRNA level was determined in the respective mutant relative to WT E. faecium LMGT 2783. In the WT strain, the *liaX* gene was transcribed at a low level, while in MUT_131, its transcription was enhanced 3-fold, reaching a relative expression level of 45.4 (Table 2), suggesting that the mutation in the UP region may have actually improved the efficiency of RNA polymerase activity. To assess whether the mutation in the UP element of the *liaX* gene also causes a polar effect on the downstream genes in its operon, and to test its effect on the expression of the LiaFSR system genes, the mRNA levels of *liaYZ* and *liaFSR* were examined. The results show that in MUT_131, the expression levels of the genes tested increased only slightly (2- to 3-fold; *liaF*, *liaR*, *liaZ*) or not at all (*liaS*), or were even reduced (*liaY*), compared to those of the WT strain (Table 2).

**TABLE 2:** Expression levels of selected genes in E. faecium wild type, MUT_131, and MUT_132^*a*^

Gene	Transcript level in:			Ratio		Function of the encoded protein
	WT	MUT_131	MUT_132	MUT_131	MUT_132	
*xpaC*	5.6 ± 1.4	ND	38.5 ± 7.1		7*	5-Bromo-4-chloroindolyl phosphate hydrolysis protein
*sgtB*	3.6 ± 0.3	ND	33.4 ± 4.6		9*	Monofunctional peptidoglycan glycosyltransferase
*liaF*	0.2 ± 0.0	0.3 ± 0.1	1.6 ± 0.1	2*	9*	Membrane-embedded regulatory protein
*liaS*	0.1 ± 0.0	0.1 ± 0.0	1.8 ± 0.4	1*	17*	Membrane-embedded bifunctional histidine kinase
*liaR*	1.7 ± 0.2	3.9 ± 0.8	20.5 ± 3.1	2*	12*	Response regulator
*liaX*	15.6 ± 3.4	45.4 ± 4.4	212.6 ± 44.0	3*	14*	Daptomycin-sensing protein
*liaY*	1.0 ± 0.2	0.3 ± 0.1	18.8 ± 4.9	0.3*	18*	PspC domain-containing protein
*liaZ*	3.1 ± 0.7	8.9 ± 2.1	69.5 ± 18.7	3*	22*	Phage holin family protein
*ef0798*	1.1 ± 0.3	ND	29.1 ± 2.5		27*	DUF1700 domain-containing hypothetical protein
*ef1533*	0.8 ± 0.2	ND	35.9 ± 3.0		45*	DUF1093 domain-containing YxeA family hypothetical protein
*ef0932*	0.3 ± 0.1	ND	12.4 ± 1.4		49*	Hypothetical protein

a Ratio values shown for the indicated mutants were calculated by dividing the relative expression level of a given mutant by that of WT strain LMGT 2783. *, statistically significant result (*P* value < 0.05). ND, not done.

### Mutation in *liaR* increases expression of *liaFSR*, *liaXYZ*, cell wall remodeling-associated genes, and hypothetical genes.

Since LiaR is a transcriptional regulator activated by diverse stressors to protect the bacterial cell (21, 22), we examined the effect of the MUT_132 *liaR* missense mutation 527C→A on the expression of selected genes. We chose homologs of genes involved in the development of resistance to antimicrobial compounds in different species, such as (i) *dxsA* and *dgkB* (L. lactis resistance to DAP, GRA, aminoglycosides, vancomycin, fosfomycin, and AurA53- and EntL50-like bacteriocins) (12), (ii) *dltABCD* (KinG-LlrG-mediated L. lactis resistance to DAP, GRA, and AurA53- and EntL50-like bacteriocins) (26), (iii) *spxB* and *oatA* (CesSR-mediated L. lactis resistance to peptidoglycan hydrolysis) (27), (iv) *xpaC* (*llmg1115*) and *spx* (*llmg1155*) (CesSR-mediated L. lactis resistance to lactococcin 972 [Lcn972]) (28, 29), (v) *mprF* (B. subtilis resistance to DAP) (30), (vi) *dltX* (Bacillus thuringiensis resistance to cationic AMPs) (31), (vii) *fmtA*, *murZ*, *pbp2*, *plsC* (*sa1548*), *sgtB*, and *spsA* (VraSR-mediated S. aureus resistance to glycopeptides and β-lactam antibiotics) (32), and (viii) *liaFSR*, *liaXYZ*, *ef0798*, *ef0932*, and *ef1533* (LiaSR-mediated E. faecalis resistance to DAP and LL-37) (22).

The *liaR* mutation did not markedly alter the expression of the majority of these genes. Of the 27 tested, 16 showed an at most 2-fold increased transcript level (*dgkB*, *dltABCDX*, *dxsA*, *fmtA*, *mprF*, *murZ*, *oatA*, *pbp2*, *plsC*, *spsA*, *spx*, *spxB*), indicating that although these genes play an important role in protection against antimicrobial compounds in other species, they appear not to be regulated by LiaR affected by *liaR* mutation in MUT_132, and this mutant-decreased sensitivity to the compounds tested in this study is not caused by the changes in the expression of these genes. However, the other nine genes assayed showed a much higher transcriptional enhancement in the LiaR mutant, ranging from 7-fold to almost 50-fold. The latter was observed for two hypothetical genes, *ef1533* and *ef0932*, expressed at a low level in the WT strain. The expression of another hypothetical gene, *ef0798*, was increased more than 20-fold, and that of all genes from both *lia* operons was increased from 9-fold (*liaF*) to 22-fold (*liaZ*). Interestingly, the *liaR* mutation upregulated the expression of the *liaFSR* operon itself 12-fold, indicating its autoregulation (Table 2). The other two modestly upregulated genes, *xpaC* and *sgtB* (7- and 9-fold, respectively), encode yet another hypothetical protein and a glycosyltransferase involved in peptidoglycan synthesis. The latter suggests an indirect involvement of LiaR in the cell wall modification in response to the presence of antimicrobials studied here.

### BHT-B-resistant mutants are cross resistant to some bacteriocins and antibiotics.

**FIG 3:**
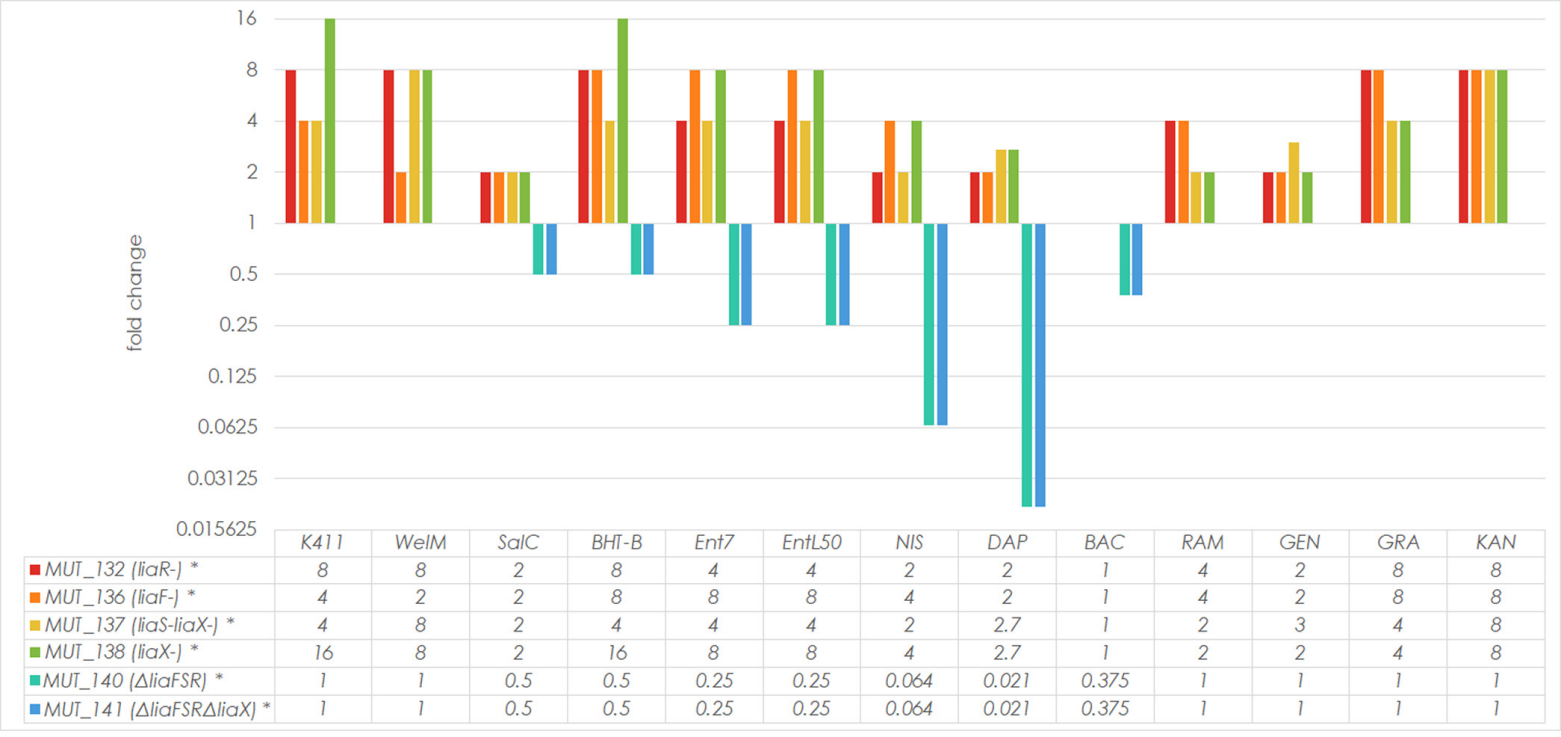
Fold change in resistance levels of adaptive and deletion mutants of E. faecium to selected bacteriocins and antibiotics. The fold change values shown for the indicated agents were calculated by dividing the MIC (μg/mL) value for a given mutant by that for WT strain LMGT 2783. A fold change of 1 indicates no change in the level of resistance between the WT and mutant, a fold change of >1 indicates a decrease in the mutant's sensitivity compared to that of the WT, and a fold change of <1 indicates an increase in the mutant's sensitivity compared to that of the WT. A logarithmic scale with a base of 2 was used on the *y* axis. *, statistically significant results (*P* value < 0.05). NIS, nisin; DAP, daptomycin; BAC, bacitracin; RAM, ramoplanin; GEN, gentamicin; GRA, gramicidin; KAN, kanamycin. Shown are only those agents to which at least one mutant strain was significantly more or less sensitive than the WT.

We chose four spontaneous E. faecium BHT-B-resistant mutants affected in four different genes (MUT_132, MUT_136, MUT_137, and MUT_138) to determine their cross-resistance to other AurA53 (K411)- and EntL50 (EntL50 [EntL50A and EntL50B], enterocin 7 [Ent7: Ent7A and Ent7B], weissellicin M [WelM], and salivaricin C [SalC])-like bacteriocins. Additionally, sensitivity to nisin was tested, as it also targets the cell envelope but has a mechanism of action different from that of AurA53- and EntL50-like bacteriocins, and the LiaFSR system has previously been shown to be involved in nisin resistance in E. faecalis (21). The mutants exhibited a 2-to 16-fold-lower sensitivity than that of WT E. faecium to all of the bacteriocins tested. The least-reduced sensitivity was that to nisin and SalC (no more than 4-fold) and the highest (16-fold) was that to K411 (Fig. 3). Thus, the extent of K411 resistance matched that to the original selective agent, BHT-B.

We then determined the cross-resistance of the four mutant strains against diverse antibiotics targeting the membrane (GRA) or various cellular processes, such as synthesis of the cell wall (DAP, amoxicillin, ampicillin, BAC, carbenicillin, cefuroxime, RAM, VAN), protein (chloramphenicol, chlortetracycline, clindamycin, erythromycin, GEN, KAN, streptomycin, tetracycline), DNA (ciprofloxacin and norfloxacin), folate (trimethoprim), and respiration and pyruvate metabolism (nitrofurantoin). The mutants showed a 2- to 8-fold lower sensitivity than the parental strain to three peptide antibiotics (RAM, GRA, and DAP) and two aminoglycosides (KAN and GEN) only (Fig. 3). The greatest decrease of sensitivity was to KAN and GRA, and the least was to DAP or GEN. Noteworthy here is the observation that all the mutants showed cross-resistance to all the peptide antimicrobials tested, with the single exception of BAC, which is a mixture of cyclic peptides (Fig. 3).

### Deletion or overexpression of *liaFSR* and/or *liaX* genes affects E. faecium sensitivity to selected antibiotics and bacteriocins.

Since in the spontaneous resistant mutants the affected LiaF, LiaS, LiaR, and LiaX proteins could still be functional in their mutated forms, we asked how a full deletion of the genes in question would impact the sensitivity to diverse antimicrobials. To this end, we constructed two strains with a deletion of the whole *liaFSR* operon (MUT_140) or of the *liaFSR* operon plus the *liaX* gene (MUT_141) and determined their sensitivity to AurA53- and EntL50-like bacteriocins, nisin, BAC, DAP, GEN, GRA, KAN, and RAM. The sensitivity to K411, WelM, RAM, GRA, KAN, and GEN turned out to be unaffected by the deletions, while that to the other antimicrobials was markedly increased in both strains. The highest increase in sensitivity was that toward DAP and nisin, 48- and 16-fold, respectively. For the remaining agents, it ranged between 2- and 4-fold (Fig. 3). It is remarkable that the effect of the gene deletion was opposite that of the spontaneous point mutations, which decreased the sensitivity to antimicrobials.

The two deletion mutants, MUT_140 and MUT_141, were then used to determine the effect of an overexpression of individual components of the LiaSR system on E. faecium sensitivity to antimicrobials. The *liaS*, *liaR*, or *liaSR* genes cloned under the strong *ptcB* promoter (P*ptcB*) were introduced into MUT_140 or MUT_141, and the sensitivity of the resulting strains (see Table 4 for details) was determined. The deletion strains carrying an empty vector served as controls. Overexpression of *liaS* alone had no effect on the sensitivity of either deletion strain, while overexpression of *liaR* or *liaSR* decreased their sensitivity to differing extents. In the strain with the *liaFSR* deletion, the overexpression of *liaR* or *liaSR* either effectively nullified the effect of the deletion, decreasing the sensitivity to the WT level (for BHT-B, Ent7, EntL50, and DAP), or even reduced the sensitivity even further, to a level comparable to that of the spontaneous point mutants (for K411, WelM, SalC, GRA, and GEN) (Table 3). In the strain with both *liaFSR* and *liaX* deleted, this effect was even more pronounced (Table 3).

**TABLE 3:** Sensitivities of *liaFSR* and *liaFSR-liaX* deletion mutants of E. faecium overexpressing *liaS* and/or *liaR* gene to selected bacteriocins and antibiotics^*a*^

Strain	Genotype	MIC (μg/mL)												
		BHT-B	K411	Ent7	EntL50	WelM	SalC	NIS	RAM	GRA	DAP	BAC	KAN	GEN
LMGT 2783	WT	3.2	3.2	1.6	1.6	6.3	25	125	0.4	0.006	3	32	32	4
MUT_143	Δ*liaFSR* pGhost9 (empty vector)	1.6	3.2	0.4	0.4	6.3	12.5	8	0.3	0.006	0.38	24	32	12
MUT_144	Δ*liaFSR* pGh:P*ptcB*:*liaS*	1.6	3.2	0.4	0.4	6.3	12.5	8	0.3	0.006	0.38	24	32	12
MUT_145	Δ*liaFSR* pGh:P*ptcB*:*liaR*	3.2	12.5	1.6	1.6	25	50	16	0.3	0.047	6	24	64	32
MUT_146	Δ*liaFSR* pGh:P*ptcB*:*liaSR*	3.2	12.5	1.6	1.6	25	50	16	0.3	0.047	3	16	64	32
MUT_147	Δ*liaFSR* Δ*liaX* pGhost9 (empty vector)	1.6	3.2	0.4	0.4	6.3	12.5	8	0.3	0.006	0.38	16	32	16
MUT_148	Δ*liaFSR* Δ*liaX* pGh:P*ptcB*:*liaS*	1.6	3.2	0.4	0.4	6.3	12.5	8	0.3	0.006	0.38	16	32	16
MUT_149	Δ*liaFSR* Δ*liaX* pGh:P*ptcB*:*liaR*	12.5	25	1.6	3.2	50	50	16	0.6	0.047	12	16	128	64
MUT_150	Δ*liaFSR* Δ*liaX* pGh:P*ptcB*:*liaSR*	12.5	25	1.6	3.2	50	50	16	0.6	0.047	8	24	128	64

a Values shown are MIC_50_s (μg/mL) (for BHT-B, K411, Ent7, EntL50, WelM, SalC, nisin, RAM, and GRA) or MICs (μg/mL) (for DAP, BAC, KAN, and GEN). Data are the mean of three biological replicates, and the standard deviation in all cases was not greater than 0.000.

## DISCUSSION

Enterococci are a large group of lactic acid bacteria that, owing to their tolerance to salts, acids, and activity against Listeria monocytogenes (33), have long been used in the production of fermented dairy and meat products (34). On the other hand, enterococci also include pathogenic strains carrying virulence factors and a considerable resistome, which makes them one of the most serious and steadily increasing causes of hospital epidemiological risks (35). The vast majority of enterococcal infections are caused by E. faecalis, which is more pathogenic than E. faecium. However, E. faecium is intrinsically more resistant to antibiotics and is the leading cause of VRE infections (23). Accordingly, the Infectious Diseases Society of America has classified E. faecium into a top-priority ESKAPE group of six highly virulent and MDR bacterial pathogens (Enterococcus faecium, Staphylococcus aureus, Klebsiella pneumoniae, Acinetobacter baumannii, Pseudomonas aeruginosa, and Enterobacter species) for which novel antimicrobial agents urgently need to be developed (36). Alternative measures, such as the use of bacteriocins, could be a potential solution, especially since several international health agencies recommend and support the development of such interventions (37). Due to their compelling advantages (broad or narrow spectrum of activity, high potency, lack of or low cytotoxicity, and possibility of bioengineering), bacteriocins could be used in preventive strategies separately or in combination with other antimicrobial agents (e.g., antibiotics) (38). In fact, several bacteriocins have already been subjected to preclinical and clinical trials, and thiostrepton (class I bacteriocin) is already used in veterinary medicine (39, 40). Nevertheless, in order to more fully exploit their efficacy in an era of rapidly developing multidrug resistance among pathogens, more basic research on the mechanisms of cell killing and the development of resistance to bacteriocins is needed. The present study fills some of the knowledge gaps regarding the genetic basis of the development of resistance to potent antienterococcal bacteriocins, pointing out the common and divergent features regarding cross-resistance to antibiotics.

Many bacteriocins act by targeting a specific receptor. Such receptor-dependent activity usually narrows down the spectrum of susceptible bacteria and additionally, through receptor mutations, may lead to the rapid development of resistance, as has been observed, for example, in the case of the mannose phosphotransferase (Man-PTS) receptor, whose inactivation induced by the presence of a bacteriocin led to full resistance of the mutants (41, 42). Therefore, the receptor-independent, membrane-directed AurA53- and EntL50-like bacteriocins, with their broad spectrum of activity against Gram-positive bacteria, may be very promising in treating or aiding in the treatment of certain bacterial infections, including those caused by pathogenic enterococci (11, 14, 26). The cytoplasmic membrane is a critical and highly conserved structure, and one would expect difficulty in the development of resistance to membrane-targeting antimicrobial compounds. However, we did observe here such a phenomenon induced by a membrane-active antimicrobial tested against E. faecium. Moreover, the mild resistance, in the range between 2- and 16-fold, triggered by a bacteriocin from the aureocin A53-like group, BHT-B, conferred a cross-resistance of the mutants to other antimicrobial agents, including not only BHT-B-related bacteriocins but also clinically relevant peptide antibiotics targeting the membrane (GRA) or cell wall (RAM and DAP) and also two aminoglycosides (KAN and GEN). Bacteriocin resistance did not change the fitness of mutants with regard to their efficiency of multiplication compared to that of WT cells, their ability to utilize different carbon sources, and their resistance to NaCl or SDS. The absence of biological costs of acquiring resistance may suggest that mechanisms other than those used to protect against AurA53- and EntL50-like bacteriocins are involved in protection against salinity and detergent stress.

Whole-genome sequencing of the resistant E. faecium mutants identified adaptive mutations in the *liaFSR* and *liaXYZ* operons. LiaFSR is a well-known regulatory system engaged in the response to cell membrane stress in Gram-positive bacteria, while the role of LiaXYZ remains poorly understood with the exception of the LiaX function as the main modulator of LiaFSR in E. faecalis (22). Previous studies have shown that the development of enterococcal resistance to DAP most commonly involves mutations in the *liaFSR* genes leading to MICs ranging from 3 μg/mL to as high as 48 μg/mL (43). The DAP MIC for the WT E. faecium strain LMGT 2783 used here was 3 μg/mL, within the 2- to 4-μg/mL range typical for most of the several thousand strains of this species isolated from European and U.S. medical centers (44). In contrast, the BHT-B-resistant mutants obtained exhibited slightly higher DAP MIC values, up to ~8 μg/mL; they were sufficiently high to exceed the susceptible dose-dependent or even resistant clinical MIC breakpoints set for DAP and E. faecium by the Clinical and Laboratory Standards Institute (CLSI) (≤4 and ≥8 μg/mL, respectively) (45). Although the occurrence of LiaFSR adaptive mutations has already been reported as a factor in the development of resistance to DAP and some other antimicrobial compounds across enterococci (21, 22, 43, 46–49), it has not been linked to the resistance to cell envelope-active peptide antibiotics or AurA53- and EntL50-group bacteriocins studied in this work, nor to aminoglycosides, which are chemically different from the above-mentioned compounds and use a wholly distinct mechanism of action.

**FIG 4:**
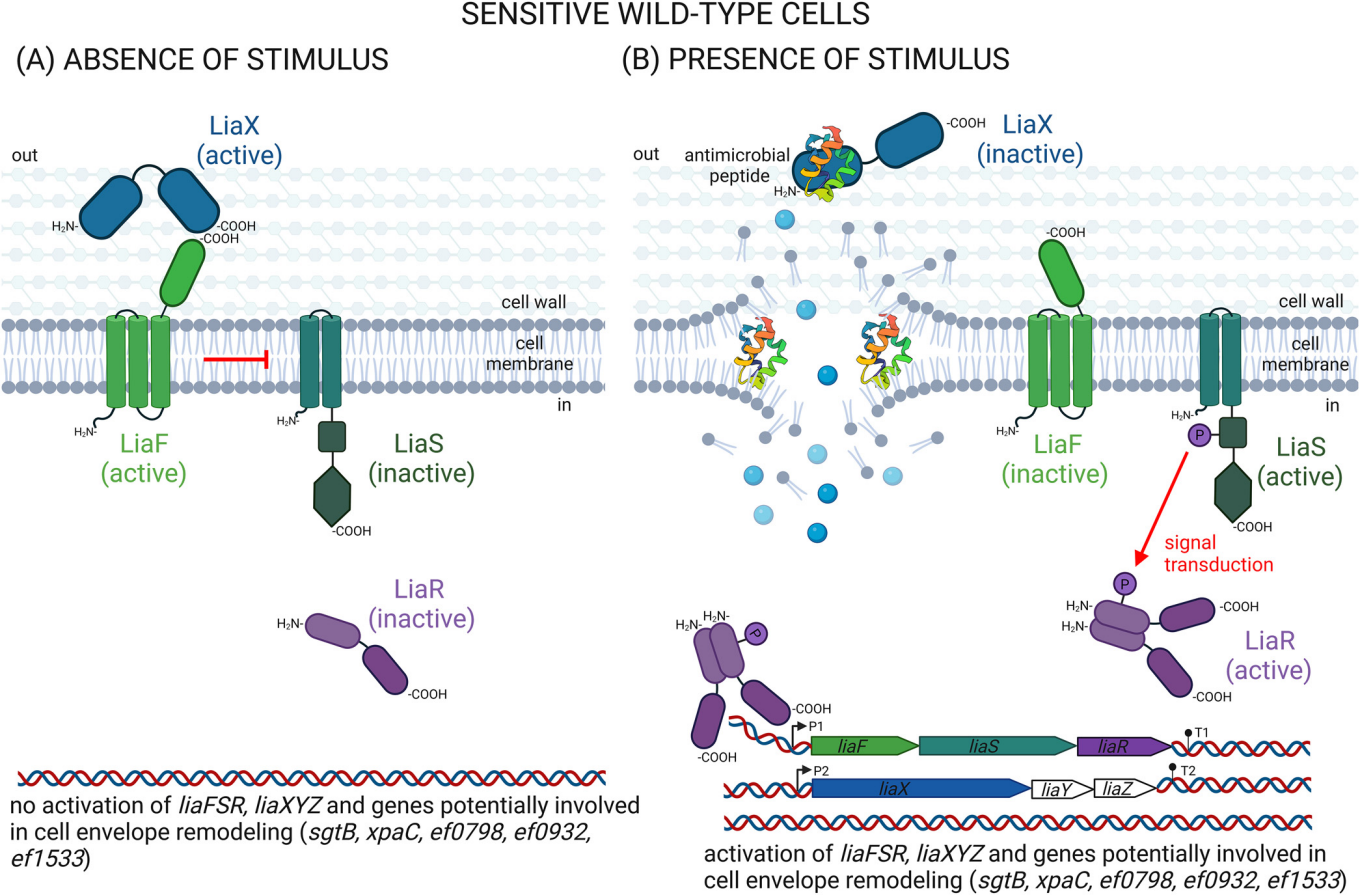

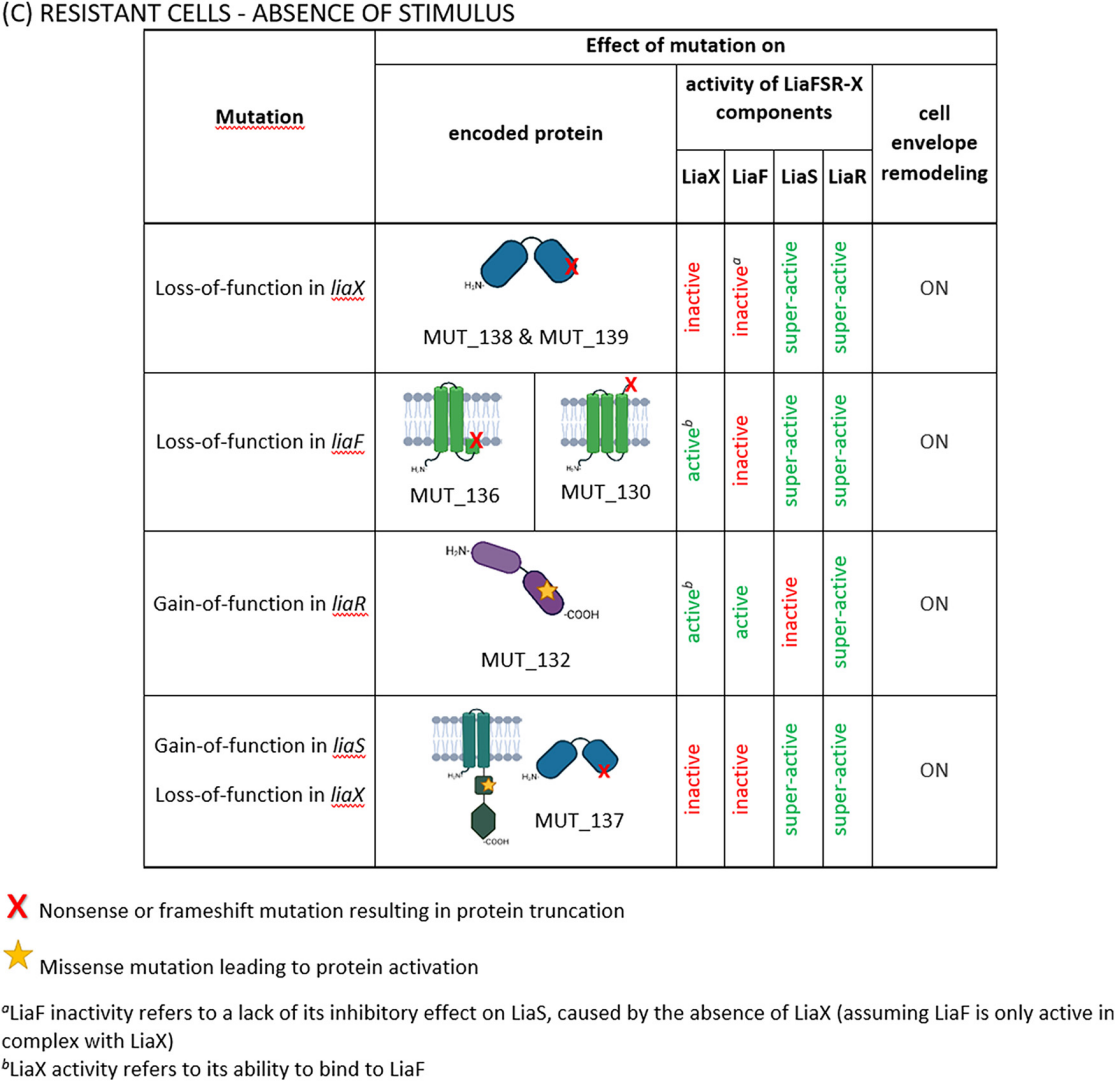
LiaFSR-X signaling in WT E. faecium and its resistant mutants. (A) In the sensitive WT cells and in the absence of an antimicrobial agent (AurA53- and EntL50-group bacteriocins, DAP, GRA, RAM, KAN, or GEN), the signaling pathway is switched off by negative interactions of the LiaXF complex with LiaS. (B) In the presence of an antimicrobial agent triggering changes in cell envelope integrity, the N-terminal domain of LiaX senses the agent by an unknown mechanism, which induces a conformational change in the C-terminal domain, releasing LiaF no longer able to inhibit LiaS. Consequently, LiaS undergoes autophosphorylation and activates LiaR by transferring the phosphoryl group to it, leading to downstream LiaR-dependent changes in the expression of its own *liaFSR* operon, *liaXYZ*, and several genes involved in protective remodeling of the cell envelope. Such activity of the system guarantees only a basal level of resistance, insufficient to protect against higher concentrations of an antimicrobial. (C) In all resistant adaptive mutants, LiaR is constitutively active both in the presence and in the absence of an external stressor, even though the activity of other components of the LiaFSR-X response pathway may vary depending on the presence of the agent. The activity of those components in the absence of the stressor is indicated. Loss-of-function mutations in the LiaX or LiaF inhibitors release large amounts of free LiaS, which efficiently phosphorylates LiaR. Gain-of-function mutation in LiaS causes its superactivation and escape from LiaFX-dependent inhibition. Gain-of-function mutation in LiaR renders it independent of LiaS phosphorylation and therefore constitutively active. The result of all these mutations is an enhanced LiaR-dependent activation of transcription of *liaFSR*, *liaXYZ*, and cell-envelope remodeling genes and thus effective protection against cell damage.

The *liaXYZ* operon identified in this work as another target for adaptive mutations is much less well understood and, until recently, was described in the literature as encoding proteins with unknown functions (previously designated YvlB-PspC-YvlD). However, following the identification of mutations in *liaX* in DAP-resistant E. faecalis mutants, its potential role in protection against this antibiotic was suggested (46, 50, 51). The final resolution of its pivotal function in E. faecalis was achieved in the work of Khan et al., in 2019, who showed that LiaX binds to DAP and thereby leads to activation of the downstream LiaFSR response pathway (Fig. 4A and B) (22). Since LiaX functions as an intrinsic modulator of this system’s response to cellular stress, it is tempting to propose that LiaFSR, usually referred to as a three-component system, is in fact more complex and together with LiaX could actually constitute a four-component LiaFSR-X system.

Using *in silico* analysis, we identified different effects of the adaptive mutations in the *liaF* and *liaX* genes encoding two inhibitors of the LiaFSR-X system (truncation of the C-terminal domains due to frameshift or nonsense mutations, e.g., LiaF^Gln115X^, LiaF^Ile64IlefsX4^, LiaX^Leu461TrpfsX20^, or LiaX^Gln507X^) in comparison with those in *liaS* and *liaR* encoding two signaling transducers (only amino acid substitutions such as LiaR^Thr176Lys^ or LiaS^Gln184Arg^ in the active sites of the respective enzymes). This suggests that the mutations in *liaF* and *liaX* could be classified as loss-of-function mutations resulting in the inactivation of the LiaF and LiaX inhibitors. In contrast, since there were no frameshift or nonsense mutations in the LiaSR signal transducers that would disable the pathway, the missense mutations in *liaS* and *liaR* could be classified as gain-of-function mutations that likely resulted in activation of LiaS and LiaR. Remarkably, although all these adaptive mutations were of a different nature (missense, nonsense, frameshift) and impact on the particular component of the LiaFSR-X system (activation or inactivation), and occurred in genes with dissimilar functions (activators or inhibitors of the LiaFSR-X response pathway), the levels of resistance to a particular antimicrobial compound were comparable among all the mutants, suggesting similar impacts of the individual components of the LiaFSR-X system on the development of resistance to this compound. Given the response cascade in the LiaFSR-X pathway, which is activated by antimicrobial peptides abolishing the inhibitory effect of LiaXF over LiaSR (Fig. 4B), it seems quite reasonable that the outcome of the adaptive mutations generated here is to decouple this system from the inducing action of the antibacterial compounds.

Thus, we propose that in the MUT_137, MUT_138, and MUT_139 strains, the effect of the loss-of-function mutations in *liaX* was an abolishment of the LiaXF-dependent inhibition of LiaS and a consequent activation of LiaR as a result of its phosphorylation by LiaS. Similarly, the loss-of-function mutations in *liaF* carried by MUT_130 and MUT_136 strains released LiaS from the LiaF-dependent inhibition, which in turn led to LiaR activation (Fig. 4C). Remarkably, inactivation of LiaX alone was sufficient to release the histidine kinase LiaS from the inhibitory effect of LiaF, which is known to be a potent inhibitor of LiaS in the absence of a stressor. This suggests that in fact, a complex of interacting LiaX and LiaF proteins is required for the LiaS inhibition and the absence of either component abolishes this inhibition. In contrast, the gain-of-function mutations in *liaS* and *liaR*, which appeared in the MUT_137 and MUT_132 mutants, respectively, led to direct activation of the encoded proteins despite the unaffected inhibitory action of the LiaX-LiaF complex (Fig. 4C). The MUT_137 strain is unusual because of its double mutation of the loss-of-function (in *liaX*) and gain-of-function (in *liaS*) types (Fig. 4C), but its resistance level is similar to that of the single mutants. This lack of additivity is consistent with the proposed mode of action of the two types of mutations.

A systematic review of the published literature for reports on the genetic alterations in DAP-resistant enterococci indicates numerous mutations in the *liaFSR*-*liaX* genes. Although highly variable in terms of localization in individual genes, they are overwhelmingly of the same type as in our study, namely, loss-of-function in genes encoding inhibitors of the system (*liaX* and *liaF*) and gain-of-function in those coding for activators (*liaS* and *liaR*) (43, 46, 47, 50, 52). This indicates a common mode of resistance development via *liaFSR*-*liaX* mutations in all enterococci, as well as potentially in other species carrying these genes. Consequently, we propose that the final effect of any adaptive mutation in the *liaFSR* or *liaX* genes leading to resistance to the compounds tested always comes down to a constitutive activation of LiaR. Thus, LiaR can be activated by loss-of-function mutations in *liaX* or *liaF*, whose products in WT bacteria constitutively inhibit LiaSR activity, or by gain-of-function mutations in *liaS* or *liaR*, which in turn cause, respectively, a corresponding increase in LiaR phosphorylation by activated LiaS or an LiaS-independent activation of LiaR by, e.g., altering its oligomerization state toward the active and phosphorylation-independent DNA-binding dimer, as has already been proposed by Davlieva et al. (53).

The dominant role of LiaR in the regulation of the cellular stress response in E. faecium is confirmed by the fact that in mutants lacking the *liaFSR* or *liaFSR-liaX* genes and thus hypersusceptible to most of the antimicrobial agents tested here, an in-*trans* delivery of multiple copies of *liaR* alone was sufficient to fully reverse the effect of the deletions to produce a WT level of sensitivity or even increased resistance of the recombinant strains. We did not observe such an effect when another gene subject to gain-of-function mutation, *liaS*, was overexpressed. This lack of a suppressive effect is consistent with the accepted mode of action of the LiaS kinase: in the absence of its substrate, LiaR, it could not effect susceptibility of the deletion mutants. Intriguingly, we have previously noted a similar paramount role of a response regulator from the four-component stress response system YsaCB-KinG-LlrG in L. lactis. Although apparently unrelated to the LiaFSR-X system, YsaCB-KinG-LlrG is also involved in protection against AurA53- and EntL50-like bacteriocins and some peptide antibiotics (26). Similarly to LiaR in the present work, the LlrG response regulator from the YsaCB-KinG-LlrG system conferred resistance even in the absence of the other components of this pathway; this activity could be triggered by gain-of-function mutation or an upregulation of its expression (26). This implies that the relevance of response regulators as the ultimate effectors of mutations in genes of an entire pathway involved in bacterial resistance to antimicrobial agents is universal across the diverse response system functioning in different genera.

Regulation of *liaFSR* transcription by activated LiaR has been described for several bacterial genera, including *Enterococcus* (17, 22, 53–55). Also, the LiaR-dependent regulation of *liaXYZ* expression has been described in some aspects in both E. faecalis and E. faecium (22, 53). In contrast, other genes regulated by LiaR, including those involved in protection against external stressors, are poorly characterized, especially in E. faecium. Here, we determined in E. faecium the effect of the Thr176Lys substitution in LiaR on the expression of *liaFSR*, *liaXYZ*, and diverse other genes known to be involved in conferring resistance to peptide antibiotics (including glycopeptides, DAP, and GRA) and bacteriocins (including AurA53- and EntL50-like groups and Lcn972) in various bacterial species (12, 22, 26–32). As expected, the mutated LiaR strongly activated the expression of the *liaFSR* and *liaXYZ* operons, their mRNA levels reaching about 10 to 20 times that found in WT E. faecium. LiaY and LiaZ are transmembrane proteins of unknown function, possibly involved in the signaling cascade as interaction partners with LiaX (22). Based on LiaY homology with the E. coli PspC protein involved in maintaining cell envelope integrity under a variety of membrane stresses (19), one can speculate about its involvement in cell membrane remodeling. An almost 10-fold expression enhancement was also observed for the *xpaC* and *sgtB* genes involved, respectively, in CesSR-mediated resistance to Lcn972 in L. lactis (28, 29) and VraSR-mediated resistance to glycopeptides and β-lactam antibiotics in S. aureus (32). CesSR of lactococci and VraSR of staphylococci are equivalents of LiaSR in enterococci, so they are likely to be subject to similar regulation, although, as indicated by the data obtained here, this is not the rule, since three other lactococcal CesSR regulon genes (*spxB*, *oatA*, and *spx*) (27–29) and five other staphylococcal VraSR regulon genes (*fmtA*, *murZ*, *pbp2*, *plsC*, and *spsA*) (32) appeared not to be regulated by LiaR in E. faecium, at least under the conditions tested here. As could be predicted from species relatedness, the strongest activation by LiaR^Thr176Lys^ was observed for the *ef0798*, *ef0932*, and *ef1533* genes, elements of the LiaR regulon in E. faecalis. These three genes are scattered throughout the chromosome and encode membrane proteins with unknown function in both species, but their regulation by LiaR suggests that they could be important for resistance development and thus they deserve further study. The EF1533 protein containing the DUF1093 domain seems particularly relevant, as it is homologous to the YxeA protein which in B. subtilis is regulated by the YxdJK TCS. This system regulates the expression of various genes involved in bacterial cell wall modifications in response to cell envelope-directed antimicrobial peptides (56–58). Similar targeting of cell envelope modifications, may be due to overexpression in the LiaR^Thr176Lys^ mutant of SgtB glycosyltransferase, which is involved in peptidoglycan synthesis (59, 60).

Last, but not least, we show here that the decrease in susceptibility of E. faecium can also be induced by merely increasing *liaX* expression alone, without a concomitant gain-of-function mutation in LiaR, which was induced by a nucleotide change that streamlines the UP sequence in the MUT_131 mutant. The *liaX* transcript was elevated only 3-fold, but this was enough to induce an 8-fold decrease in the mutant's susceptibility to BHT-B. This is an ambiguous effect insofar as one would assume that the more LiaX inhibitor, the stronger the inhibition of LiaSR and thus the increase in the mutant's susceptibility, but in this case, we noted the opposite effect. However, Khan et al. observed a similar phenomenon of protection against DAP of susceptible E. faecalis strains induced by the addition of exogenous LiaX or N-terminal LiaX (22). It is suggested that the protective effect of LiaX is dependent on the presence of LiaR and that LiaX does not sequester DAP just to prevent its binding to the cell. Our study of *liaFSR* and *liaXYZ* transcripts in MUT_131 does not provide a firm conclusion regarding the effect of increased *liaX* expression on the participation of individual gene products in the increased susceptibility of this mutant, as the resulting mRNA levels of the individual genes were not increased at all or were increased only slightly (2- to 3-fold maximum). However, it is tempting to speculate that the 2-fold increase in *liaR* expression in the MUT-131 mutant is sufficient to confer resistance and that the encoded protein is the receiver of the signal from overproduced LiaX, which then passes it on to genes involved in protective remodeling of the cell envelope. In agreement with this hypothesis is the fact that in E. faecalis, the addition of DAP and exogenous LiaX or N-terminal LiaX induces a significant increase in the DAP MIC with only a small increase in *liaR* expression but that *liaR* deletion prevents the acquisition of resistance (22). In this case, further research is needed to elucidate the mechanism of LiaR activation induced by excess LiaX in *Enterococcus* spp.

For a long time, bacteriocins were believed not to induce the emergence of resistance among susceptible bacterial strains. However, here we show that in the presence of AurA53- and EntL50-like bacteriocins, the susceptibility of E. faecium can be reduced due to the emergence of adaptive mutations in the LiaFSR-X response system; moreover, these mutations confer resistance to certain membrane-active peptide antibiotics, but mostly not to those targeting cell wall synthesis or intracellular structures. A systematic analysis of the obtained mutations in the individual components of LiaFSR-X indicated that despite their diversity, their ultimate effect was the activation of the response regulator LiaR, which then strongly and constitutively activated genes engaged in protective cell envelope remodeling, leading to cell resistance. It seems likely that similar mechanisms can function in other species, including those using nonorthologous systems of response to extracellular stressors. Given the unique relevance of LiaR and possibly also its counterparts in the development of resistance, one should consider designing inhibitors of their activity to be administered together with membrane-acting agents to limit this deleterious phenomenon.

## MATERIALS AND METHODS

### Bacterial strains, plasmids, and culture conditions.

The bacterial strains and plasmids used are listed in Table 4. E. faecium LMGT 2783 and its spontaneous (MUT_130 to MUT_132, MUT_136 to MUT_139) or deletion (MUT_140 and MUT_141) mutants were grown in brain heart infusion (BHI) medium (Oxoid, UK) at 37°C. E. faecium MUT_142 to MUT_149 carrying pGhost or pGh:P*ptcB* were grown in M17 medium (Oxoid) supplemented with 0.5% glucose (GM17) and 5 μg/mL erythromycin (Em). For the expression of genes cloned under the *ptcB* gene promoter (P*ptcB*), the bacteria were cultured in M17 medium supplemented with 1% cellobiose (CM17) and 5 μg/mL Em. Soft agar and agar plates were prepared by adding agar (Merck, Germany) to 0.75% and 1.5%, respectively.

**TABLE 4:** Bacterial strains and plasmids used in this study

Strain or plasmid	Description	Source^*a*^ (reference)
Strains		
Enterococcus faecium LMGT 2783	Indicator strain, host strain	LMGT
Enterococcus faecium LMGT 2783 mutants		
MUT_130, MUT_131, MUT_132, MUT_136, MUT_137, MUT_138, MUT_139	Spontaneous mutants obtained in the presence of BHT-B	This study
MUT_140	LMGT 2783 with *liaFSR* deletion	This study
MUT_141	LMGT 2783 with *liaFSR* and *liaX* deletions	This study
MUT_142	MUT_140 carrying pGhost9	This study
MUT_143	MUT_140 carrying pGh:P*ptcB:liaS*	This study
MUT_144	MUT_140 carrying pGh:P*ptcB:liaR*	This study
MUT_145	MUT_140 carrying pGh:P*ptcB:liaSR*	This study
MUT_146	MUT_141 carrying pGhost9	This study
MUT_147	MUT_141 carrying pGh:P*ptcB:liaS*	This study
MUT_148	MUT_141 carrying pGh:P*ptcB:liaR*	This study
MUT_149	MUT_141 carrying pGh:P*ptcB:liaSR*	This study
Plasmids		
pGEMT	Amp^r^, M13*ori*, linear T-overhang vector	Promega
pGhost9	Em^r^, *repA*(Ts)	INRA (66)
pIBB-JZK	Amp^r^, Tet^r^, cellobiose-responsive promoter (P*ptcB*)	IBB PAS (67)

a Strains or plasmids derived from the collections of the Laboratory of Microbial Gene Technology, Department of Chemistry, Biotechnology and Food Science, Norwegian University of Life Sciences, Ås, Norway (LMGT); the French National Institute for Agriculture, Food and Environment, Jouy en Josas Cedex, France (INRA); and the Regional Strains and Plasmids Collection of the Institute of Biochemistry and Biophysics, Polish Academy of Sciences, Warsaw, Poland (IBB PAS).

### Bacteriocin and antibiotic preparation.

Chemical synthesis of bacteriocins BHT-B, K411, Ent7, EntL50, WelM, and SalC with a purity of >95% was performed by PepMic (People’s Republic of China). Nisin (≥900 IU/mg) was purchased from Glentham Life Sciences Ltd. (UK). Stock solutions (1 mg/mL) were prepared by dissolving lyophilized bacteriocins in 0.1% trifluoroacetic acid (TFA; Sigma, Germany). Nisin solution was additionally filtered through a 0.22-μm filter (Millipore, Germany). Gramicidin (GRA; Sigma), ramoplanin (RAM; Sigma), carbenicillin (Millipore), and chlortetracycline (Millipore) were purchased in powder form. Their stock solutions were prepared in ethanol (GRA, 1 mg/mL) or in sterile water (RAM and chlortetracycline at 1 mg/mL, carbenicillin at 100 mg/mL). Other antibiotics used in this study were purchased in the form of antibiotic-impregnated strips (bioMérieux, France; except for bacitracin [BAC], which was from Liofilchem, Italy).

### Selection of spontaneous BHT-B-resistant mutants.

Spontaneous resistant mutants were obtained by growing E. faecium LMGT 2783 in the presence of the bacteriocin as described previously (41). The MUT_130 to MUT_132 mutants were obtained at a BHT-B concentration of 20 μg/mL, while MUT_136 to MUT_139 were obtained at a BHT-B concentration of 22 μg/mL. The stability of changes in BHT-B sensitivity was determined by continuously subculturing mutants in BHI broth without bacteriocin. Subcultures were formed at 24-h intervals, and sensitivity to BHT-B was validated after 1, 5, and 10 passages with simultaneous control of culture purity.

### Determination of susceptibility to bacteriocins, antibiotics, NaCl, and SDS.

The sensitivity of the mutants to AurA53- and EntL50-like bacteriocins, nisin, RAM, GRA, carbenicillin, and chlortetracycline was determined using microtiter plates with serial 2-fold dilutions of the antimicrobials, as described previously (41). The lowest concentration of the agent reducing visible bacterial growth by at least 50% was taken as the MIC (MIC_50_) value. The sensitivity to other antibiotics was determined using agar plates with antibiotic strips as described previously (12). The antibiotic concentration at the point where the inhibition zone intersected the strip was taken as the MIC value. The antibiotic susceptibilities of E. faecium LMGT 2783, MUT_130 to MUT_132, MUT_136 to MUT_139, and MUT_140 and MUT_141 were tested in ISO-BHI medium (Oxoid) supplemented with 5 μg/mL Em when appropriate, while those of E. faecium MUT_142 to MUT_149 were tested in CM17 medium supplemented with 5 μg/mL Em. To study the effects of NaCl and sodium dodecyl sulfate (SDS) on bacterial growth, saturated overnight cultures of E. faecium were used for inoculation at a dilution of 1:100 in BHI medium with serial dilutions of these compounds at concentrations of 0.5 to 10% (wt/vol) and 3.0 × 10^−2^ to 2.5 × 10^−3^% (wt/vol), respectively. Bacterial cultures were incubated at 37°C without shaking for 24 h in a Bioscreen turbidometric automated analyzer (Bioscreen C, Growth Curves Ltd., Finland) in Honeycomb 100-well plates (Oy Growth Curves Ab Ltd.). MIC_50_ values of NaCl and SDS were defined based on bacterial growth kinetics as the lowest concentration of a compound at which at least 50% inhibition of strain growth was observed. The assays were made at least in triplicate.

### DNA isolation, manipulation, and data analysis.

PCR products or DNA fragments from agarose gel were purified with the Wizard SV gel and PCR clean-up system (Promega, USA). Plasmid and genomic DNA was isolated using a plasmid minikit and a genomic minikit (A&A Biotechnology, Poland). For DNA manipulation, restriction and modification enzymes purchased from Thermo Fisher Scientific (USA) were used. Samples for genome sequencing were prepared with the Nextera XT DNA sample preparation kit, the Nextera XT indexing kit, and the PhiX control V3 kit (Illumina, USA) according to the manufacturer’s instructions and sequenced on a MiSeq Sequencer (Illumina). The data were analyzed with CLC Genomics Workbench 8.5 (Qiagen, Germany). Nucleotide sequences were translated to corresponding peptide sequences using an online translation tool on the ExPasy server (61). LiaF and LiaS transmembrane helices were predicted using TMHMM Server v. 2.0 (62). Conserved domains (CD) and active sites of LiaFSR-X were identified using the CD search online tool at the NCBI Conserved Domain Database (63). Additionally, LiaX N- and C-terminal domains composed mainly of α-helices and β-strands (22), respectively, were predicted based on the structure modeled using the I-TASSER web service (64). Protein models were visualized using Protter (65).

### Quantitative analysis of gene expression.

mRNA levels of genes of interest were determined by reverse transcription-quantitative PCR (RT-qPCR) in relation to the *purM* and *tuf* genes encoding, respectively, phosphoribosylaminoimidazole synthetase and elongation factor Tu. Bacterial pellets were collected from at least three independent cultures in the midexponential growth phase (optical density at 600 nm [OD_600_] of 0.8) as described previously (26). Total RNA was extracted using the GeneMATRIX universal RNA purification kit (EURx, Poland), and first-strand cDNA was obtained using random primers with the RevertAid first-strand cDNA synthesis kit (Thermo Fisher Scientific) in accordance with the manufacturer’s instructions. Finally, qPCR was performed using gene-specific primers (Table 5), and data were analyzed using the modified Δ*C_T_* (cycle threshold) method as described previously (26) with suitable modifications for the amount of the cDNA template in the reaction mixture (for each gene, three amounts of cDNA, 63, 21, and 7 ng per well, each in duplicate, were used).

**TABLE 5:** Primers used in this study

Primer	Nucleotide sequence (5′→3′) and restriction site^*a*^
For cloning	
1224/1233	CGCCAGGGTTTTCCCAGTCACGA/AGCGGATAACAATTTCACACAGG
pGhfor/rev	TGTAAAACGACGGCCAGTG/AGTACCGTTACTTATGAGC
pIBB-JZKfor/rev	AGTCGCCTAAAGGTTGC/CGATGTTCTGTCCCTTG
LiaFSRUPfor/rev	GCCTGATGGAGATGAAGAAGAG/ CAGGATCCGCAGTGCTTCGACTACC
LiaFSRDNfor/rev	CAGGATCCCAAGCAGCTATCTATGCG/ CCGTAAGTTTCTGGCAG
LiaFSROUTfor/rev	CGTAGCAGATACGAGCAC/TCTGGCTGTTCAACTGAC
LiaXfor/rev	GCATTGTCGGTCATAGG/GCTCACATGGACCATCG
LiaXOUTfor/rev	GCGGTACGCAAGGTATG/AGGGCAGGGACAGTATC
LiaSRfor/rev	GCTAGGATCCACCTTGGTTGGAGATGTC/CGAT**CTCGAG**GATTATCCTCTTTATCATCG
LiaSrev	CGAT**CTCGAG**CTTTATCTATGCCTCCTTC
LiaRfor	GCTAGGATCCCAAGGTCCCTTTGATG
LiaXfor/rev	GCTAGGATCCGCTTAAGGAGGCAATTTCC/CGAT**CTCGAG**CTCAAGGCTGAACATCTC
pGhLiaXfor/rev	CTCAAGATGGAGTAAAAG/CTCAAGGCTGAACATCTC
For RT-qPCR	
EfDgkBaF/EfDgkBaR	CCAAGAATGAAGCACGTAGAG/CGGAGCGATTCCATTAACC
EfDxsAaF/EfDxsAaR	CAGTCAATGCAGCGATACC/GCAGAAGCGAAAGTGATAGAG
EfDltAaF/EfDltAaR	TGCCGATTGAAGAGCATAC/GACAGGAACCGAAGGAATATC
EfDltBaF/EfDltBaR	TGGGTATATGGCATGGATTG/CAACTGCTCTTTATGCTTCTTC
EfDltCaF/EfDltCaR	GTCGAGCTAGACGGAGAG/ATGCAGTTGCTTGTTGAATG
EfDltDaF/EfDltDaR	ATACGTTGACGGCGATTC/CTCTCTCTTCAGCATGTTCC
EfDltXaF/EfDltXaR	ATGAACAAATGGCGTTCTAATC/ACAGATAAATCAGGAACAGCAG
EfMprFaF/EfMprFaR	AGCGGATGTTTGCCTATG/CCAGAAGGATCACCCATTAC
EfSpxBaF/EfSpxBaR	ACGACGCCATGAAACATC/TGCTAGGTACCAGCTATCAG
EfOatAaF/EfOatAaR	CCAAGGCACAAGGGAATAAG/CGACATCTCCATCTACCACTAC
EfXpaCaF/EfXpaCaR	CGCCATGATCCAGTCAAAG/TTCGGCAGATGCGTATAAAG
EfSpx_bF/EfSpx_bR	ACCATCAAATGCCTTACAGAG/CGTTTAGCTAATAGATCGTCAAATC
EfLiaFaF/EfLiaFaR	CATCGATTTGGGCAATACAC/CTCTAAACGAATAGCCACTCC
EfLiaSaF/EfLiaSaR	GACTAGCCGTCCTCAATATG/TGAAGCTCACGAGCTAATC
EfLiaRaF/EfLiaRaR	TAATCAGGAAATCGCGGATG/GCTTGAGTTCGGTCTTCTAC
EfLiaXbF/EfLiaXbR	GGAGCCAGATTGAGGTTAATG/CGTGGTCGTATACTCGTTTAG
EfLiaYaF/EfLiaYaR	TCTGCACGAACAAACTATGG/GTCACTCCATTCGTCATCATC
EfLiaZaF/EfLiaZaR	GGTCCATGTGAGCAGTATTTG/TAAAGGGCAGGGACAGTATC
Ef0798aF/Ef0798aR	TCTCCTGTCCTCATTCCAC/GCCAACACCTAAGATACTGATAC
Ef0932aF/Ef0932aR	CAAAGAACTGGCGGATGAC/GCAAGGAGCCAGCTATAAAC
Ef1533aF/Ef1533aR	TCCCTGGTATACTTGATCAGC/TTGGCATCAGCAGCTTTC
EfPlsCaF/EfPlsCaR	GAGAGTGACAGTGCGTTTC/GTGTTCGTCGTTCGATCTC
EfFmtAaF/EfFmtAaR	CCGAAATGAATTAGGCACAGG/GCCGCTGTAAGGATGTTTATC
EfPbp2bF/EfPbp2bR	CTATTCGGATCATCGGTTCTG/CACTAGTGGAGAAGAAGGAAAG
EfMurZaF/EfMurZaR	CGAATGGGCGCTGATATTAC/CTGCCAATAGTCCAGCAATC
EfSgtBaF/EfSgtBaR	GGAAGGGACAAGTGGAAATG/GGCATCTGCTACACTAAATCC
EfSpsAaF/EfSpsAaR	CAAGAGCTAACGGGACAATC/TGATCGTGCCAAACGAAC
EfPurMbF/EfPurMbR	CAATATGGGCATCGGTATGG/TCTTTGGCGATCACTTTCC
EfTuf_cF/EfTuf_cR	GCCTAGCAAATCCTCAAGAC/TGTGCGGTATTGATCGTAATC

a BamHI restriction sites are underlined, and XhoI restriction sites are in bold.

### Construction of deletion mutants.

The *liaFSR* and *liaX* genes were deleted using homologous recombination. First, plasmids harboring DNA fragments flanking the *liaFSR* or *liaX* genes were constructed. DNA fragments upstream (UP) and downstream (DN) of *liaFSR* were amplified with primer pairs LiaFSRUPfor/rev and LiaFSRDNfor/rev, respectively (Table 5). Purified PCR products were digested with BamHI and ligated with T4 DNA ligase. To amplify the joined flanking fragments, an additional PCR was performed using suitable UPfor and DNrev primers. The PCR product was purified and cloned into pGEM-T Easy (Promega) by TA cloning and then into pGhost9 using ApaI and NotI. A region encompassing the *liaX* gene was amplified using LiaXfor/rev primers (Table 5), purified, and cloned into pGEM-T Easy by TA cloning. The *liaX* coding sequence was then cut out using HindIII and OliI, single-stranded overhangs were blunted using Klenow fragment of DNA polymerase I, and the plasmid was religated with T4 DNA ligase. Finally, the insert containing *liaX* flanking regions was cloned into pGhost9 using ApaI and NotI. Primer pairs 1224/1233 and pGhfor/rev (Table 5) were used to confirm the presence of correct inserts in the pGEM-T Easy and pGhost9 plasmids, respectively. To force a double-crossover recombination between the plasmids and the chromosome, E. faecium LMGT 2783 culture harboring the construct with the *liaFSR* flanking fragments and E. faecium MUT_140 culture harboring the construct with the *liaX* flanking fragments were diluted 10^3^-fold in BHI medium containing Em (5 μg/mL) and incubated for 1.5 h at 30°C and then for 3.5 h at 37°C. To select integrants, cultures were streaked on BHI agar plates supplemented with Em (5 μg/mL) and incubated at 37°C. To cure the integrants of the plasmids, cultures were grown in the absence of antibiotics at 30°C. The deletion of *liaFSR* and *liaX* was confirmed by colony PCR with LiaFSROUTfor/rev and LiaXOUTfor/rev primers pairs, respectively (Table 5).

### Construction of expression mutants.

The pGhost9 vector was used for expression of the *liaS*, *liaR*, *liaSR*, and *liaX* genes. First, the genes were amplified using primer pairs LiaSRfor/LiaSrev, LiaRfor/LiaSRrev, LiaSRfor/rev, and LiaXfor/rev, respectively (Table 5). Purified PCR products were cloned into pJZK-IBB under the cellobiose-responsive promoter P*ptcB* using BamHI and XhoI, excised together with P*ptcB* using PstI and NotI, and cloned into pGhost9. The obtained constructs, pGh:P*ptcB:liaS*, pGh:P*ptcB*:*liaR*, and pGh:P*ptcB:liaSR*, were expressed in E. faecium MUT_140 and MUT_141. Due to the toxicity of cloned *liaX* to the bacterial host, it could not be expressed under P*ptcB* in any of the deletion mutants. Therefore, we attempted to clone *liaX* gene under its native promoter in pGhost9, but again we could not obtain a LiaX-expressing strain (results not shown). To confirm the presence of correct inserts in the pGEMT-T Easy, pIBB-JZK, and pGhost9 plasmids, primer pairs (Table 5) 1224/1233, pIBB-JZKfor/rev, and pGhfor/rev were used, respectively.

### Data analysis.

All results were developed using Excel (MS Office Standard 2016) for Windows. Standard deviations (±) of the dispersion of the data relative to the mean were compiled using results from at least three biological replicates. Statistical analysis to compare the means of the data of two groups were performed using the *t* test. Results were considered statistically significant if the probability value (*P* value) was less than 0.05.
